# From Catastrophe to Hope: Hunters' Experiences in the Eradication of Sweden's First African Swine Fever Outbreak

**DOI:** 10.1155/tbed/6407552

**Published:** 2025-08-27

**Authors:** Hedvig Gröndal, Hedvig Stenberg, Karl Ståhl, Susanna Sternberg Lewerin, Erika Chenais

**Affiliations:** ^1^Department of Animal Biosciences, Swedish University of Agricultural Sciences, Uppsala, Sweden; ^2^Swedish Veterinary Agency, Uppsala, Sweden

**Keywords:** ASF, community, focus group discussion, thematic analysis, wild boar, wildlife management

## Abstract

In September 2023, Sweden experienced its first ever outbreak of African swine fever (ASF). One year later, in September 2024, Sweden was declared free from ASF. One of the first actions taken toward control and eradication was an intensive search for wild boar carcasses. This was mostly carried out by local hunters. After some time, a core area of infection was fenced in, and all remaining wild boar in that area were culled. Local hunters also performed the culling. This paper presents results from a qualitative study, drawing on focus group discussions (FGDs) with the hunters involved in identifying and managing the ASF outbreak. The aim of the study was to take a transdisciplinary approach in exploring the experiences of the hunters, specifically focusing on their motivation for engaging in the outbreak management. The interviews were analyzed using thematic analysis. The study gives important insights that can be utilized in case of future outbreaks among wildlife: engaging the hunters appears to be facilitated by their local connectivity, involving commitment to the hunting grounds and to their local society. In addition, local knowledge about the land and wildlife seems to have facilitated effective carcass searching and culling. It is, therefore, of importance for authorities to establish relationships with local hunters and to acknowledge the hunters' pivotal position in managing an outbreak. Our study shows that it is essential for the hunters to feel that they are not governed in detail, but that they have some flexibility to take decisions based on their specific local knowledge. Further, our results show that it is important to acknowledge the social aspects of searching and culling work in order to engage hunters. These kinds of social activities is a prerequisite for effective management.

## 1. Introduction

In September 2023, Sweden experienced its first ever outbreak of African swine fever (ASF), as a wild boar, found dead by a local hunter in a rural area 170 km northwest of Stockholm, was confirmed positive for the ASF virus (ASFV) [1]. One year later, in September 2024, Sweden was declared free from ASF. Upon detecting the outbreak, strict control measures were put in place in a defined, so-called infected zone. Restrictions initially included a complete ban on all access to forest areas in the infected zone. As the epidemiological situation improved, restrictions were progressively lifted and finally completely removed. One of the first actions taken toward control and eradication was an intensive, active search for wild boar carcasses undertaken mostly by local hunters. At first, the hunters were volunteers, but they were later remunerated. After some time, the core area of infection was fenced in and all remaining wild boar in that area were culled. Local hunters also carried out fence maintenance and culling. The Swedish Board of Agriculture (SBA) is the competent authority for animal health in Sweden and, consequently, the agency taking decisions concerning ASF outbreak management, for example. Part of an ASF expert group at the Swedish Veterinary Agency (SVA) gave expert advice to the SBA and representatives from the Swedish Association for Hunting and Wildlife Management (SJF) were also involved in the planning and execution of these measures.

The outbreak in Sweden was part of the global ASF epidemic that has been ongoing in Europe since 2007 and in the EU since 2014, affecting both domestic pigs and wild boar [2, 3]. As with domestic pigs, ASFV infection in wild boar typically results in severe clinical disease with high case fatality rates [4]. In Northern and Central Europe, the infection has mostly spread among wild boar. These animals have, therefore, been the main victims of the disease as well as the culprits in disease spread [5, 6]. The outbreak in Sweden only affected wild boar. Controlling ASF in wild boar populations has proved difficult. In the current epidemic, so far only Belgium, the Czech Republic, and Sweden have managed to regain freedom from the disease after its introduction into a wild boar population [7, 8].

Research from affected countries in Europe has highlighted the important role of local hunters in the early detection, surveillance, and control of ASF in wild boar [9]. Several studies have investigated aspects of hunters' participation in ASF surveillance and control, aiming to better understand how their engagement can be sustained using tools from the veterinary application of participatory epidemiology [10, 11]. Specifically, investigating hunters' perceptions of the surveillance and control measures applied during outbreaks, studies with hunters in Germany, Estonia, Latvia, and Lithuania come to similar results: control measures that hinder hunting (e.g., a hunting ban, hunting restrictions, and fences) and those regarded as not abiding by the ethical charter of fair hunting practices (e.g., shooting females) are generally perceived negatively and as ineffective for controlling ASF [12–15]. These studies further show that local hunters generally have a high trust in their own organization's capacity of ASF control and that of local governmental agencies, but a low trust in central government agencies [12–15]. Compensating hunters for their work and consulting hunters in the design of control measures to avoid measures being negatively perceived are emphasized as important to maintain hunters' engagement in ASF control, especially in countries with prolonged disease presence [11, 16].

The Swedish ASF situation was radically different from the situation in the Baltic States and in Germany. Estonia, Latvia, and Lithuania have been continually infected since 2014 and Germany since 2020, whereas the Swedish outbreak was eradicated in less than 12 months [1, 7, 17, 18]. These different time scales and sizes of outbreaks will probably impact how the hunters perceive the disease, the control measures, and the agencies that take and implement decisions and restrictions. In addition, the way that the ownership of forests, wildlife management, and hunting is organized differs greatly between countries. Although some aspects of how hunters experience ASF outbreaks can be generalized, many relations and situations will be context specific and, therefore, necessary to investigate in each infected country or setting. A previous study analyzed the attitudes and practices of Swedish hunters toward reporting and sampling wild boar carcasses alongside hypothetical ASF control and surveillance measures in a preoutbreak situation [19]. The hunters in that study were generally positive about participating, but voiced some concerns regarding how their participation would be organized in an outbreak situation and how much work could be done on a voluntary basis during an outbreak.

Hunting is deeply rooted in Swedish society [20, 21] and to a high degree accepted by the public [22]. Hunting in Sweden needs to be understood in the context of “allemansrätten”—a universal right of access to nature [21]. This specific right is stated in the individual rights of Swedish foundational law and embedded within Swedish culture. As such, it structures the public's relationship with nature as something everyone has the right to access, but also as something everyone has a responsibility toward [23–25].

A large proportion of Swedish hunters are members of the Swedish Association for Hunting and Wildlife Management (SJF). SJF is described by Essen and Tickle [26] as having “a semigovernmental mandate,” including a public mandate (and funding) for certain wildlife management services [26] ([27, 28]). Further, SJF is a strong lobbying organization, which together with other Swedish hunting associations not only function as forums where hunters can have their voices heard, but also have “a de facto impact on policy” [29] (p. 123). Importantly, Swedish hunters' engagement in national hunter organizations and local hunting teams needs to be understood in relation to a national tradition of a strong civic society with a high degree of membership in social movements and interest organizations [30], of which hunter associations are, thus, just one example [31, 32]. Studies shows that, in an international perspective, civic society in Sweden is strong and vital [33] even in rural areas [34]. Strong civic societies have been described as associated with a high level of social capital in terms of trust [33]. Notably, a strong civic society has been described as crucial for collective action in case of crisis, in particular in rural areas [35]. Moreover, in Sweden, there is a specific long-term cooperation between the public and the authorities (in practice between hunters and the SVA) for the surveillance of disease in wildlife. In 1945, a health monitoring program for wildlife was initiated in Sweden. The program was sponsored by SJF, the Swedish Environmental protection agency, and the Swedish government, led by the SVA. The program is still running, with the engagement of hunters and close cooperation between the hunters, hunting organizations, and the authorities being identified as success factors [36].

Previous research shows that hunting in Sweden is of a collective character and has a social function [21, 37, 38]. Drawing on interviews with Swedish hunters, Von Essen and Allen [39] state that these hunters describe themselves as more knowledgeable and concerned about wildlife and ecology, as well as of statutory regulations, than hunters in other countries [29, 39]. The hunters describe hunting as a social glue in rural communities, reproducing a “Scandinavian way of living” where closeness to nature as well as being law abiding is valued. However, Essen and Allen mention that narratives on closeness to nature can be identified among hunters in other cultural contexts as well. International studies show how care for wildlife management and hunters' understanding of themselves as wildlife stewards tend to be tied to the identity of a “local” hunter—a hunter connected to the local community and the surrounding land. The identity of the local hunter is further connected to seeing hunting not only as a hobby, but as a way of life [40].

While SJF has close connections to the Swedish government, studies also show that Swedish hunters are critical of the state and its agenda. Several studies have shown how Swedish hunters describe the state as prioritizing conservation (in particular regarding wolf policy) instead of rural economics and social welfare, and that they perceive national wildlife policy as being governed by a distant elite [41, 42]. Swedish hunters tend to be skeptical about centralized and detailed regulation, and instead favor “autonomy to pursue its own affairs and police within its own ranks according to its own moral compass” [29] (p.145−155).

Transdisciplinary research placed in the social interface between hunters, wild boar, ASF, outbreak management, and control restrictions, which is needed for a deeper understanding of the situated factors governing the motivation and decision-making processes for hunters during outbreaks, is scarce. The aim of this study was to take a transdisciplinary approach in exploring the experiences of the hunters directly involved in managing the Swedish ASF outbreak, with a specific focus on the hunters' motivation for engaging.

## 2. Materials and Methods

This qualitative interview study included focus group discussion (FGD) with hunters who had participated in the management of the ASF outbreak in Sweden.

### 2.1. Participant Selection

Hunting teams were recruited with support from a regional representative of the SJF. We aimed for hunting teams in the area who had been active in the search and culling of wild boar in connection with the ASF outbreak, both within and outside the core area of infection. Five hunting teams were recruited. Three of the recruited groups had their hunting grounds within the core area and two of the groups had their hunting grounds outside, but close to, the core area. To create inclusive discussions, we asked for five to seven members from each hunting team to join an FGD. However, in the end, only three to five members from each team took part (total *n* = 20, 5 FGDs). All participants were adult men. One participant, who was a member of two hunting groups, took part in the discussions with both groups and, consequently, appears twice in the data.

### 2.2. Data Collection and Analysis

The FGDs took place in Fagersta and were conducted in Swedish. The discussions were led by a social scientist (Hedvig Gröndal), with support from a veterinary epidemiologist (Hedvig Stenberg or Erika Chenais). The discussions lasted between 1.5 and 3 h, with a 30 min break included. A topic guide with open-ended questions (Supporting Information 1) was used to initiate discussions and to make sure that the relevant subjects were covered. The topic guide contained questions about hunting before the outbreak, experiences from engagement in the outbreak, and the future. The participants were also invited to reflect on matters relevant to them beyond the topic guide.

Consent was obtained from all participants. The discussions were recorded and later transcribed. A thematic analysis was conducted. This is a common approach in social science research aiming at identifying reoccurring themes in interviews [43]. After several readings of the transcripts, one of the researchers (Hedvig Gröndal) performed an inductive coding of the interviews. This means that patterns were identified in the transcribed interviews and that the initial codes developed were very closely linked to the actual quotes. In the second step, codes were clustered into broader themes. At this stage in the thematic analysis, all authors discussed the broader themes together and reiterated the analysis until the final broader themes presented here emerged. One of the broader themes was divided into subthemes. The quotes were translated into English after the analysis and should be seen as illustrations of the text.

## 3. Results

Eight broader themes were distinguished during the thematic analysis: “Hunting before the outbreak: lifestyle, passion for the forest, and responsibility for the land,” “The outbreak—almost the end of the world,” “Engagement as a way to cope and take responsibility,” “Social relationships and status strengthened,” “The importance of the local: knowledge and rootedness,” “About the authorities and the organization of searches,” “Challenges,” and “The future—hopefulness.” The themes are presented and developed in this section of the manuscript.

### 3.1. Hunting Before the Outbreak: Lifestyle, Passion for the Forest, and Responsibility for the Land

Hunting was described as a central part of the participants' lives and, in several FGDs, expressed in terms of a lifestyle, and by some participants as “life.” Hunting was portrayed as comprising much more than just hunting, containing a passion for spending time in, and caring for, the forest. One hunter expressed this clearly:

Hunter 4: All of us were in the forest. It is our life, after all (FGD 1).

To hunt was described as including care for the specific hunting ground and of the wildlife living there, not only when actively hunting:

Hunter 2: It becomes a lifestyle entirely. You live in this every day. And we, what's the word, we're out on the ground, refilling bait, we're looking after the land (FGD 2).

As illustrated by this quote, the hunters identified themselves as stewards of the hunting ground and this stewardship involved practices of taking care of the land, and, for example, managing baiting stations. In some teams, the hunters were inspecting the grounds every day, carefully observing all human and wildlife activity. In one FGD, the participants emphasized that they hunted on the same land as their parents and grandparents, and they described how this created a specific sense of responsibility for, and connection to, their hunting ground.

In all the FGDs, the social aspects of hunting were described as important. The hunting teams organized social events outside of hunting, but the participants also described the hunting per se as a social experience. One example is given below:

Hunter 1: We're not saying that we are dependent on the hunting, but it's like smoking, drugs, everything. You get addicted to hunting. It's like, I've been hunting for 47 years I think I've counted it to. […]

Hunter 3: There's spiritual care in it. Like it's, both physically and mentally, it's a breathing space, those days you have [hunting] in the year (FGD 2).

In the quote above, hunting is described as important for the hunters' physical and mental wellbeing, and as creating “breathing spaces,” in contrast to other parts of their life. The hunter ambiguously states that hunting, on the one hand, is not an addiction, but, on the other, that it is, comparable to drug use and smoking—very hard to abstain from, since it creates a physical desire.

Wild boar, or the major game species that was being hunted, was described as important in three of the FGDs. These teams hunted with bait and spent a lot of time managing the baiting stations. They described hunting wild boar as an exceptionally exciting form of hunting. In two FGDs, hunting wild boar was instead described as a peripheral part of their hunting.

Notably, the FGDs who described hunting wild boar as central had their hunting grounds situated close to the municipal waste collection center that turned out to be at the epicenter of the outbreak [1]. These participants described the wild boars' access to the waste collection as having led to an immense local increase in the wild boar population, facilitating wild boar hunting. The animals' access to the garbage had worried the hunters and, in several FGDs, participants described that they had been in contact with the municipality and asked for a fence around the waste collection center, but without any actions being taken.

Hunter 2: Right, so we've had incredibly good wild boar hunting.

Hunter 3: And that probably had a lot to do with the big baiting set-up there at the garbage station (FGD 3).

As in this quote, several groups jokingly described the waste collection center as a large “baiting station.” While this on the one hand, as mentioned above, had worried them, they also acknowledged that the center had led to an increase in the wild boar population in the area. This had facilitated their hunting of the species. The waste collection center thus appears to have an ambiguous position for the hunters—it was seen as risky, but also as providing hunting opportunities.

### 3.2. The Outbreak—Almost the End of the World

Participants in one of the FGDs described how they had noticed that something was wrong with the wild boar in their hunting ground and that this had prompted them to send in samples to the SVA from a wild boar found dead in the area.

Hunter 3: But it was like then more and more reports came in that people had seen pigs [wild boar] behaving very strangely. […] What the hell, have they been eating fermented fruit or glycol or what could they have ingested? But that was the one [which became the first ASF-positive case]. […]

Hunter 2: Yes, I thought like hell, something is damn crazy. Why? (FGD 3).

The hunters in this team described how they had discussed the strange observed behavior of wild boar, thinking that the animals had been infected or poisoned by something in the garbage. They had, however, not suspected ASF.

In all the FGDs, the outbreak of ASF was described in terms of being a catastrophe for the hunters themselves, for the community, and for local industries. The participants described the restricted access to the forest as being particularly hard to live with. One FGD compared the situation with spending time in jail. The area had previously suffered from forest fires and some participants described themselves and the community as especially hard hit by bad luck.

Hunter 1: And it's like when you're married and your partner or the person you're married to disappears. Then, you become very lonely. That's probably the case for most people here as well (FGD 2).

Hunter 4: It was almost as if the whole world came to an end for a while when people found out that now we can't go into the forest.

Hunter 1: And pick mushrooms and walk the dogs (FGD 1).

As illustrated in these quotes, the restrictions on hunting and spending time in the woods were described with dramatic metaphors, such as separating from a spouse, spending time in prison, and the end of the world. In several FGDs, the participants further described how the situation with the outbreak was hard to manage not only for the humans in the community, but also for the hunting dogs. The dogs were described as very important for the hunters' identities and sometimes as extensions of themselves—when the dogs suffered from the loss of hunting, the hunters suffered as well.

### 3.3. Engagement as a Way to Cope and Take Responsibility

All participants had, to a varying extent, been engaged in the search for wild boar carcasses and some had been involved in the culling of wild boar. Many of the participants had been very active, taking time off from their normal work to spend up to half their days in the management of the outbreak. To engage with and act on the outbreak was described as a way of coping with the feelings of powerlessness and disaster described above.

Hunter 2: Yes, no, but it has been. We have had things to do, but like. Really that we have lost both forestry, hunting, everything. So, that has felt pretty hopeless. And then, at least I feel, or well, there are probably more than me, who have felt quite powerless. Yes, but, well, what do we do about this then? We do the best we can. And as many people as possible then, out into the woods and run around. So that we can put an end to this at some point (FGD 1).

Hunter 1: […] But for me it was that you felt that you were doing something. And it felt good to like, I don't know how to describe it. I feel proud (FGD 3).

In several FGDs, the participants stated that their engagement primarily stemmed from a desire to make the area free from ASF in order to be able to spend time in the forest and hunt again, and to get their “life” back. Several participants emphasized that engagement was a way of taking responsibility, of “doing your part,” and as a matter of self-respect, and that participating in managing the outbreak made them feel proud.

Hedvig Gröndal: But how come there has been such a commitment then?

Hunter 3: I think it's needed. Because it's something that you want. You want it to end quickly. To like do as much as you can in some way so that you can get back to what you like about being in the forest and hunting. That's really the main goal for me anyway.

Hunter 2: And it's like this is something we haven't asked for. Nobody has done that. But, now we have to deal with it. And I feel like [name], that it's an interest for me. Once we start hunting, I want to be able to stand tall in some way. Yeah, I've done everything I can do, yeah.

Hunter 3: To try to, yes, get our life back as soon as possible (FGD 3).

The participants, moreover, described how they were motivated by their desire to help out in their local community, including the local forestry and agriculture. One group described coming back to hunting as of less importance compared to helping the local industry. Participants, furthermore, said that managing the outbreak was probably facilitated by the special sense of community in this rural area with a long local history of mining and metals industry.

Hunter 2: […] Maybe it's lucky in some way that it happened here of all places. You live in a small town with its own spirit. Everyone pitches in (FGD 4).

In one FGD, the participants emphasized how the motivation to engage increased when they realized the potential consequences of the outbreak on the national level.

In some FGDs, participating in the management of the outbreak was described as making the loss of hunting easier, since the hunters could meet and spend time in the forest during searches and other disease control activities.

Hunter 3: […] Now we've been together at least.

Hunter 1: Yes, we have been walking a lot.

Hunter 3: We've had a good time anyhow, without it costing ammunition.

Hunter 2: And we grilled sausages (FGD 1).

As described above, the engagement meant that the social aspects of hunting (which are explored in detail in the next section) could be maintained. The hunters also described how the engagement also meant that, despite the restrictions, they could actually spend time in the forest.

Hunter 2: Yeah, like and then, yeah, now we're doing this instead of hunting this weekend maybe. I don't know.

Hedvig Gröndal: It was a bit of a substitute for not being able to hunt.

Hunter 2: Maybe not a substitute, but at least a reason to be out in the forest in some way (FGD 3).

As illustrated in the quote above, the searches were, therefore, not described as a substitute for hunting, but as making it possible for the hunters to spend time in the forest and with each other even when hunting was not allowed. In this way, searches appear to have served as an alternative activity when hunting was not possible.

### 3.4. Social Relationships and Status Strengthened

In several FGDs, the participants stated that the carcass searches had been positive for the social relationships within the hunting team and for the relationships between hunting teams. One FGD mentioned that they had spent more time together during the outbreak than normally and that this had tied them together. The social aspects of the search, such as joking together and having an open fire barbecue at break times, were described as important, making the physically and psychologically hard work of the search easier.

Hunter 1: I would say this as well, that what it has done, it's a bit like this has made, we have become more tightly knit, just not for the hunting, but now there is this other thing we're going to carry out. Like in a different way. When we hunt, it's like, the focus is on that. Now the focus has been on something completely different for a while. But we've still got to work together. […] (FGD 1).

The notion that the team had become more tightly knit during the restrictions and control work reoccurred in several groups. It was also expressed that managing the outbreak had strengthened the relationships between hunting teams.

Hunter 1: Yes, you get to know more people around you. We've been in the same group all the time and then there are people you haven't really known before, who haven't been out with us.

Hedvig Gröndal: From other hunting teams?

Hunter 1: Yes, that's it.

Hunter 3: Yes, mostly it's that you get to know people from around (FGD 5).

Several participants described feeling appreciated by their local community and that the status of hunters had increased due to their engagement in managing the outbreak.

Hunter 3: I think, I think our status has been raised a bit.

Hunter 2: Yes, definitely, I think so too. People have really appreciated what we have done (FGD 3).

As illustrated in this quote, the outbreak was described as raising the hunters' status and several participants described how their work had been acknowledged by the local community. In several groups, the hunters described how nonhunting locals had thanked them for their engagement and turned to them when they wanted to know things related to the outbreak and the restrictions.

### 3.5. The Importance of Local Knowledge and Rootedness

In all FGDs, the participants stressed the importance of local knowledge and rootedness for managing the outbreak. The participants said that external actors would not have been able to manage the outbreak as effectively as they had themselves.

Hunter 4: But they did talk about bringing in a group of experts with experience of eliminating raccoon dogs. But I can say with one hundred per cent certainty that they would never have succeeded as quickly as we did (FGD 1).

This quote illustrates the hunters' confidence in that their local knowledge had been crucial for effective searches and culling. A specially trained raccoon group would, according to these hunters, not have been as successful as them. The participants described how they are familiar with the specific terrain and where the wild boar usually reside. The participants also described how it was emotionally important for them to work in their own hunting grounds.

Hunter 2: But the advantage we have here is that we can find our way in the woods. We know, we know almost, well not every single stone, but we do know the way. That has to be a great advantage.

Hunter 4: And we know where the pigs [wild boar] are.

Hunter 2: Yes, that as well. Exactly.

Hunter 1: Yeah, and know the paths and […] (FGD 1).

Some nonlocals and nonhunters voluntarily participated in the searches and the hunters described their own local knowledge as being crucial to make use of this external help in an efficient way with the local hunters supervising the nonlocals and distributing the work tasks and search areas.

Hunter 2: Yes, knowing how to do it. Because I see now when you've been out searching and you've had 10 people with you from other places. Then it's easy, you can sort of explain to them, this lake and this, you'll walk here and then you can report back to me (FGD 3).

The local knowledge, connections, and networks also meant that information could move quickly through informal contacts. This was described as important for the effective culling of the remaining wild boar toward the end of the outbreak. As in the quote below, a hunter was informed by a neighbor about a wild boar in the core area, which had been assessed as free from wild boar at the time.

Hunter 1: Well it was like, just to give you an example, we thought we were rid of pigs [wild boar] in the core area, but a neighbor came to my house and knocked on the door, and said: “Hey, I saw a pig down there in the field walking around.” “It's not supposed to be there,” I said. So, we went there, […] So, we went down there and I said, well, it was pig tracks [in the snow]. Then, I called [the leaders of the hunting team] and he sent you and [name] out there, and then it was shot down, one-and-a-half, two hours afterwards (FGD 1).

The importance of hunters' local rootedness for managing the outbreak was further emphasized. The organization of hunting in this area of Sweden, where people generally hunt close to their homes and, thereby, feel long-term responsibility for their hunting grounds and wildlife management, was described as facilitating the hunters' engagement in the outbreak. Likewise, it was described how in the opposite situation, that is an outbreak in an area with few local hunters, it would be hard to engage people.

Hunter 1: Just play with the idea that [inaudible], that it's just paid hunts. You get to buy [the hunting rights], go there, and pay for a day or a weekend. And then you're gone and completely absolved of responsibility. Then, you'd be in trouble. There wouldn't be this bunch of people who go out, like local residents, who search. So, the way we hunt here in Sweden was probably, or how it is organized, and [inaudible], that's probably really what… (FGD 4).

Along these lines, some participants said that it had been more difficult to engage hunters in some hunting grounds where the hunters lived far away from the area and, consequently, lacked local connectivity except for the rented hunting ground.

Hunter 1: Yes, plus that, most people, as you said, most people live here so they can participate in the search. I mean, we know hunting teams, in this management area that [name] is talking about. Where it's just been, where maybe nobody lives in the community. And the hunting grounds of those hunting teams have been more difficult to get searched. Because it's, people don't travel up from Stockholm [the capital of Sweden] or wherever to be involved in searching the land up here (FGD 1).

The participants took pride in their identity as local hunters—knowing the land, the wildlife, and being rooted in the local community. This pride, and their conviction that they were the most competent actors to manage the outbreak, seems to have fueled their engagement in the control work.

### 3.6. About the Authorities and the Organization of Search Work

In several FGDs, the participants described the authorities' work with the outbreak and the organization of the search work in positive terms. They expressed that they had been given sufficient information and that representatives from the authorities had been available to answer their questions. Several groups referred to specific individuals from the authorities to whom they had spoken in person or listened in the large meetings arranged by the authorities to organize the searches.

Hunter 1: I don't think it was, well, in relation to how messy it could have been, I'm impressed that it worked out the way it did (FGD 3).

The participants described the reimbursement from the authorities for the time spent on the searches as adequate. One FGD described how the authorities had given the hunters flexibility and autonomy to plan the details of the searches, and that this was seen as positive and had facilitated the work.

Hunter 3: Right, but then we are all probably different. I think it feels pretty good because there has been decentralized order giving. We have had some control within the hunting team. Like we've been given areas to walk, but there's no one, who, like we've solved this based on our conditions and our, well, the crew we've had. Right, and we walk the easiest way in the terrain, so you can do it in different ways and that, it's great that we have been given that, that discretion. That no one draws the map for how to walk because then it could go a bit backwards (FGD 1).

It is also possible that the autonomy produced a sense of trust from and toward the authorities, and that this enforced the hunters' engagement. One FGD was, however, very critical about the authorities' work. The participants described a lack of leadership, especially in relation to the first days of the outbreak.

Hunter 2: No, because I perceived it as, it was absolutely not clear who, which organization, takes the lead on this. […] There was nothing, what was wrong was that no one knew who was going to make the decisions. So, one hopes they have learned the lesson on that now. There, you could say that if it hadn't worked so well at the ground level, it wouldn't have worked. So, you could say that the authorities haven't done a very good job, damn it (FGD 4).

In some FGDs, the participants had not been reimbursed for the fee paid to the landowner for the hunting rights, despite the restrictions preventing them from hunting. These groups expressed criticism toward the government and the authorities, who, in their view, should have compensated them for the fee. Some participants felt that the government had let them down in this regard.

Several improvements in relation to future outbreaks were suggested. Some participants proposed that the authorities should have a stockpile of equipment, for example shovels and four-wheel motorbikes. Several FGDs described the initial organization where the hunters themselves had to take samples from the carcasses found during the searches, as being too demanding and suggested that samples should only be taken by veterinarians. Some participants suggested that the cell phone application (WeeHunt) used in the searches should have been provided for free by the authorities. Several FGDs talked in particularly positive terms about the involvement of SJF in the outbreak.

### 3.7. Challenges

#### 3.7.1. Social Tensions

Even though the outbreak was described as strengthening the social relationships within and between hunting teams, there were also contrasting descriptions. Some participants described that they were disappointed in individual members of their hunting teams, who had dropped out of the group during the outbreak, and that this caused conflicts. In some FGDs, disappointment was expressed in members, who did not engage in the work with searches, and participants stated that this probably would negatively affect the sense of fellowship in the hunting team in the future.

Hedvig Gröndal: […] Was it difficult to get people in the hunting team to join in and volunteer or not?

Hunter 2: Yes, yeah.

Hunter 3: I suppose you could say that. There are a lot of people you're disappointed in (FGD 5).

The control work effect on the social relationships within the teams was, consequently, contradictory. It both strengthened and weakened these relationships. In a similar vein, disappointment was expressed in other hunting teams due to their lack of engagement. Thus, although the control work was described as having positive effects on relationships between hunting teams, it was also described as having negative effects when other teams were not as engaged as expected. Moreover, one FGD described its disappointment in not being fully included in the management of the outbreak during the initial phase.

While, as described above, the hunters generally expressed that they felt appreciated by the local community, one FGD also mentioned that they had been blamed for the outbreak by nonhunting locals.

Hunter 4: I think there has been a lot in Fagersta anyway. There have been accusations that it is the fault of us hunters that it is like this and…

Hunter 3: It's really difficult.

Hedvig Gröndal: With the swine fever?

Hunter 4: Yes, that it's […].

Hedvig Gröndal: But do you also feel that you have been accused somehow?

Hunter 4: Yes, I think so. By some, that there are accusations.

Hunter 3: It could be anyone.

Hunter 4: We've wanted this many pigs [wild boar].

Hunter 2: And that we don't shoot enough (FGD 1).

Only one team described this experience, but it is important to acknowledge, since it could have a negative influence on engaging hunters in future outbreaks. Moreover, a recurrent theme in several FGDs was the experience of being viewed as contagious by hunters in other parts of Sweden. As a result, several participants explained that they had been banned from hunting in other areas of Sweden and this was described as very frustrating.

Hunter 2: […] But we ourselves are banned from coming to hunt on other lands. Because we have the plague. When it broke out, the two of us were welcome to come down to Blekinge and hunt down there. But when the time came, no, they are infected with plague, there won't be any. So, it was canceled.

[…] Now, after the outbreak, the people from here are also infected with plague. So, it will be even more difficult.

Hedvig Gröndal: Have you others experienced this too?

Hunter 1: You've been invited along until you're asked: where do you live? In Fagersta. The plague? Yes. No, we are sorry, they say.

Hunter 3: Yes, it has become a bit like that.

Hunter 2: [talking at the same time] Yes, it was like we were all infected with the plague. You didn't dare say where you came from. I travel around and work all over Sweden. Now I tell people where I come from, I could hardly do that at that point (FGD 4).

This experience of being treated as contagious is problematic for several reasons. It was described as adding to the burden already inflicted on the concerned hunters and prevented any relief and a solution to the problem of not being able to hunt (on their own hunting grounds). In summary, these experiences could reduce hunters' engagement, a risk that should be acknowledged in relation to future outbreaks.

#### 3.7.2. Culling—A Necessary Evil

Some of the participants had been active in the culling of wild boar. The culling was described in terms of a necessary evil. It was emphasized that culling differed from the usual hunting. For example, the participants described that they planned the shots less carefully during the culling compared to normal hunting. Some participants had been involved in culling wild boar caught in traps and this was described as especially difficult. As in the quote below, in one FGD the culling was described in terms of “slaughter” and not as hunting.

Hunter 2: It won't be hunting really now, it almost feels like, well it's an obligation.

Hunter 4: Slaughter (FGD 1).

In some instances, participants described it as emotionally difficult to eliminate the wild boar population in the area. As described above, for three of the groups hunting wild boar was expressed as a very important part of their hunting and these groups had put a lot of effort into baiting. As an illustrative example, one FGD described the forest as having become “sterile.” The culling was described in the following way:

Hunter 2: It's a bit like sitting on a branch when sawing it off. It feels a bit like that. But if that's what we're going to do, then that's what we'll do (FGD 3).

Moreover, to cull and destroy healthy animals, and to hunt without being allowed to use the meat was described as problematic by some participants.

Hunter 2: And that, I can tell you, if [name] didn't feel that way, when I, when you go from [place] and there are two pigs [wild boar] under there and you can't take the meat home. Then, you cry inside. And we have done that. Like for every pig we've shot and it's all just going to Fagersta [to be destroyed]… (FGD 2).

Although the culling was, to some extent, described as involving problematic aspects, the participants generally stated that they accepted that it had to be done.

Hedvig Gröndal: Yes. What was that like?

Hunter 4: Right, it was an experience. Of course, it's no fun standing there killing healthy animals. But someone has to do it. I think it worked well (FGD 1).

Consequently, although the hunters felt that the culling had been hard, they seemed confident that it was necessary to be free of ASF.

### 3.8. The Future—Hope

When asked about the future, the sense of responsibility for preventing future outbreaks varied. While some participants described that they felt such a responsibility, others expressed it as impossible for them as hunters to hinder future outbreaks.

In general, the future was described in positive terms. As exemplified in the quote below, experiences gained from managing the outbreak were, to some extent, described as positive for future hunting.

Hedvig Gröndal: That's right. You've almost got together more.

Hunter 2: Yes, we've gotten together more now than we have during the hunting season [all agree] […].

Hunter 3: No, it's nice, if you say like, I've always found this a good crew and it's even better now, really.

Hunter 5: And it's becoming more close knit.

Hunter 4: But we became more close knit with neighboring teams I think now.

Hunter 5: Yes, it became much more so.

Hunter 4: The day we get to hunt it will probably work damn well (FGD 1).

Even though some worries were expressed, the participants generally stated that they believed the area would be free of ASF in the following fall, allowing them to hunt on their ground again.

Hedvig Gröndal: How do you see the future? What are you thinking? What do you think will happen? How do you think it will be?

Hunter 2: I firmly believe that the restrictions will be lifted and it will be as usual in September.

Hunter 1: I'm hoping for moose hunting then.

Hunter 2: I think we'll start hunting again then and return to normal. And then we can hope that this has brought positive things. That the moose population has increased a little bit. […].

Hunter 1: We see some light at the end of the tunnel. And I live on the conviction that there is…. October, when we're back in the forest. And then this has become a story and a lesson (FGD 4).

When the interviews were conducted, the area had not yet been declared free of ASF, but the hunters were confident that AFS was gone.

## 4. Discussion

This study explored the experiences of hunters directly involved in managing the ASF outbreak in Sweden, with a specific focus on their motivation for engaging.

The participants described hunting as very important for them—as a lifestyle, comprising much more than the actual hunting and involving a passion for spending time in, and caring for, the forest and the hunting grounds. Their descriptions included aspects of long-term responsibility for the hunting grounds and the wildlife there, in line with previous research describing Swedish hunters identifying themselves as wildlife stewards [29]. As a consequence of the extraordinary place hunting had in the participants' lives, the ASF outbreak and its impact in the form of loss of access to the forest and to hunting were depicted as a catastrophe. Engagement in the searches and the outbreak control efforts seemed to be a way of coping with this disaster, serving to give the hunters their life back again. A sense of care and responsibility for the local community was described as motivating for engaging in the outbreak management efforts. Along with previous research describing local connectivity as important for the construction of a hunter identity [40], here the hunters' self-identification as “local hunters” seemed significant for their engagement. Illustrating this, the participants explained that it had been difficult to engage hunters who lacked this local connectivity. Moreover, the hunters described how their local knowledge had been necessary for success in the search and culling activities, explicitly stating that nonlocal hunters would not have been as successful. The strong connectivity to the hunting ground and to the local community, thus seemed to facilitate the mobilization of hunters in managing the outbreak. Importantly, the participants further explained that managing the outbreak was facilitated by the special sense of community characterizing this rural area with a long local history of mining and metals industry, a desire to “do your part,” and norms that encourage individuals to take responsibility for common goods. It is also possible, however, that the engagement was assisted by the more general public understanding, prevalent in Sweden, that the forest is a shared resource that citizens care about and feel a responsibility for [23–25].

Although the hunters who participated in the searches were (after some time) remunerated, the motivators for participating (or, at the start of the outbreak, actually volunteering) might be compared to the motivators discussed for volunteering. Strong social capital and networks, as well as norms, and social trust have been shown to be positively associated with volunteering [44–46]. The hunters described that they in general felt appreciated by the local community during the outbreak, that is that their social capital increased and this was probably also a factor that contributed to their engagement [45]. Hunting was, moreover, described as a social activity, in line with previous research [37, 38]. This social aspect of the hunting likewise seemed to have been important for the hunters' engagement, as has been discussed for other types of voluntary work [47]. The searches were described as social and as involving joking and barbecues, for example. This was also described as making the hard work easier. The participants said that the search work mainly had a positive influence on the social relationships within and between hunting groups. However, the outbreak also affected social relationships negatively. The participants described being disappointed in team members and other hunting teams who had not engaged in the control work. In this sense, the outbreak had contradictory effects on social relationships—both strengthening some, but weakening others.

While studies from other countries have shown that hunters tend to have a low level of trust in the national authorities' capacity to control ASF [48], the dominant pattern in this study was that the participants described the authorities' work with the outbreak in positive terms and that hunters were prone to engage and cooperate with the authorities. This is partly surprising given previous studies showing that, as in many other countries, Swedish hunters are also skeptical toward the state and, in particular, to detailed top-down regulation [29, 41, 42]. Importantly, also in other national contexts, hunters highly value their hunting, see themselves as wildlife stewards, and care about their hunting land as well as their local communities [40]. These factors cannot, therefore, explain the participants' positive attitudes toward the authorities and their positive engagement. Several other, partly intertwined, factors might, however, have contributed to the participants' positive attitudes toward the authorities and these, in turn, fueled their positive engagement: (1) Swedish citizens generally tend to have high level of trust in public agencies [49]. (2) Hunting in Sweden is part of a strong and vibrant civic society, and engaging in voluntary work is common [31–34]. (3) The close cooperation between the authorities and SJF during the outbreak, in particular with the local representatives of this organization, facilitated trusting relationships between the hunters and the authorities. (4) The outbreak management benefited from the established relationship between the Swedish hunters and the SVA stemming from the long-term system for monitoring diseases in wildlife [36]. (5) The participants felt that their local knowledge and expertise was acknowledged by the authorities, and that they had been trusted to use this in planning the details of the search work. This translated into experiences of avoiding unnecessary state regulation [29]. (6) The hunters appreciated specific details of the outbreak management, like frequent open meetings for all hunters (and others for the general public), where representatives from authorities gave information and were available to answer questions, and made individual representatives from the authorities visible as people. This appears to have contributed to the sense of trust in the authorities. (7) The geographical location of the outbreak meant that very few domestic pigs needed to be culled—it is possible that extensive culling of domestic pigs would have provoked negative attitudes; and (8) at the time when the FGDs were conducted, the management of the outbreak appeared to have been successful and the outlook for the future was positive—the participants believed that they would soon be able to hunt again. If this had not been the case and the restrictions had been prolonged an additional year or two, the attitudes toward the authorities would probably have been much more negative. In summary, both the positive attitudes toward the authorities and, in turn, the mobilization of the hunters were probably facilitated by all these different factors in combination with the hunters' general engagement in hunting, their local connectedness, their care for their hunting ground and the wildlife in it, and the local community. As such, the engagement probably stemmed from a multitude of context-dependent factors and it is impossible to identify one factor as more important than another.

One FGD, however, expressed harsh criticism and said that they had lacked leadership from the authorities. Some groups were also critical of the state not reimbursing them for hunting ground fees. This also meant that they had been let down by the state. These are important points to consider in future outbreaks. It is also important to consider the hunters' experiences of being treated as contagious by hunters in other parts of Sweden. To be able to hunt in other areas was described as a way to lessen the burden, making hunting possible despite the restrictions. The experiences of being treated as contagious could, thus, reduce hunters' engagement, a risk that should be acknowledged in relation to future outbreaks. Risk mitigation could be envisaged in the form of information campaigns directed at the hunting community about ASF spread, virus survival, and the biosecurity measures put in place to prevent spread from the infected zone via, for example, people participating in searches and culling.

The culling was, in some instances, described as challenging and, in one group, as slaughter rather than hunting. The importance hunters pay to following the ethical chart of fair hunting practices even during outbreaks and the impact it might have on the engagement of hunters if authorities' control decisions break this chart have been previously discussed [50]. However, in this study, even though the participants described the culling as challenging, they also stated that they accepted that it had to be done.

The study had some limitations. The participants generally described that they had been more active in managing the outbreak than other members in their hunting teams and it is probable that this affected their experiences. Consequently, the hunters interviewed had actually had access to the forest and to the social aspect of their hunting community despite the restrictions, and it is possible that this positively influenced their attitude toward the authorities. The strengths of the study can primarily be attributed to its transdisciplinary methodological approach. Outbreaks of animal diseases are complex events, involving not only pathogens, animals, and a specific natural environment, they also involve humans and as such they are also social events, and need to be studied as such. By using transdisciplinary research teams, with expertise in both epidemiology and social science, detailed and multifaceted knowledge on animal disease outbreaks can be acquired—knowledge that is much needed in order to manage the complexities of these kinds of events.

Taken together, this study gives some important lessons that can be utilized in case of future outbreaks. The engagement of the hunters appears to be tied to their local connectivity, involving commitment to their specific hunting land and to their local society. In addition, local knowledge about the land and wildlife seemed to have facilitated effective carcass search and culling. It is, thus, of importance to establish relationships with local hunters and to acknowledge their pivotal position in managing an outbreak where wild animals are affected. In case of an outbreak in an area where there are no local hunters (e.g., with large parts of the hunting grounds being rented out to external hunters), there must be strategies in place that compensate for the lack of this valuable resource. The findings in this study, however, indicate that it is of importance to engage local actors in established local networks [35]—even when there are no local hunters, and to make use of local key persons who are trusted in this process. We, moreover, argue that the sense of mutual trust between authorities and the people working on the ground with the outbreak is necessary for effective disease management, regardless of which kind of actors (hunters or others) are involved in disease management. Large meetings for all involved where questions and criticism toward the authorities' outbreak management can be posed and where authorities emerge as individuals probably facilitate such trust. In addition, it seems important for the people working with searches as well as culling to feel that they are not governed in detail, but that they have some flexibility to take decisions based on their specific local knowledge.

Our results further show that it is important to acknowledge the social aspects of search and culling work for hunters' engagement. Therefore, these kinds of social activities should not be viewed as a peripheral part of outbreak management—there needs to be space for them. If hunters feel that there are benefits from participating in outbreak control, they will probably be more prone to engage.

## Data Availability

The data that support the findings of this study are available upon request from the corresponding author. The data are not publicly available due to privacy or ethical restrictions.

## Supporting Information

Supporting Information Supporting Information 1: Topic guide for the discussions.
