# Supplementary material for: From Catastrophe to Hope: Hunters' Experiences in the Eradication of Sweden's First African Swine Fever Outbreak
Source: Transbound Emerg Dis. 2025 Aug 27;2025:6407552. doi: 10.1155/tbed/6407552 (PMC12408132; doi:10.1155/tbed/6407552)
Supplement: Supporting Information — 1: Topic guide for the discussions. [file 6407552.f1.docx]

# Additional File 1 – Topic guide for group discussions

## BEFORE

Can you describe your hunting team?

Can you describe your everyday wild boar hunting situation before the outbreak?

Before the AFS outbreak, what did you do if you found dead wild boar? (did it ever happen?)

What were your thoughts about African swine fever before September 6^th^ 2023?

## AFTER

And what are your thoughts about ASF now?

Do you remember how it all started?

In what way have you been involved in the management of the outbreak?

Why did you engage?

How was it to engage?

- Were you prepared? If/If not – how?
- What has been working well and what has been the challenges? (Something that was hard? Was it clear what you were supposed to do and how to do it?)
- Hunters that were involved in the culling: ask specifically about this.

How has the outbreak, the management of it and the related restrictions influenced your life?

What is your experience from the authorities’ work with ASF? (In general and concretely in relation to your engagement)

## THE FUTURE

What do you think about the future for wild boar and the hunting of wild boar after the outbreak? Have you changed your way of hunting? Has your view of wild boar changed? How?

Do you, as hunters, feel responsible for hindering future ASF outbreaks? Why/Why not?

Can you, as hunters, do anything to prevent future ASF outbreaks in Sweden?

What is most central for you (risks for the wild boar, risks for pig farmers, societal risks?
